# Development in a Dish—*In Vitro* Models of Mammalian Embryonic Development

**DOI:** 10.3389/fcell.2021.655993

**Published:** 2021-05-25

**Authors:** Yasmine el Azhar, Katharina F. Sonnen

**Affiliations:** Hubrecht Institute, KNAW (Royal Netherlands Academy of Arts and Sciences) and University Medical Center Utrecht, Utrecht, Netherlands

**Keywords:** embryonic development, *in vitro* model system, stem-cell-based embryo-like models, pluripotent stem cells, blastoids, gastruloids, organoids

## Abstract

Despite decades of research, the complex processes of embryonic development are not fully understood. The study of mammalian development poses particular challenges such as low numbers of embryos, difficulties in culturing embryos *in vitro*, and the time to generate mutant lines. With new approaches we can now address questions that had to remain unanswered in the past. One big contribution to studying the molecular mechanisms of development are two- and three-dimensional *in vitro* model systems derived from pluripotent stem cells. These models, such as blastoids, gastruloids, and organoids, enable high-throughput screens and straightforward gene editing for functional testing without the need to generate mutant model organisms. Furthermore, their use reduces the number of animals needed for research and allows the study of human development. Here, we outline and discuss recent advances in such *in vitro* model systems to investigate pre-implantation and post-implantation development.

## Introduction

Embryonic development describes the establishment of the body plan and all organs within an organism. Precise control of numerous processes, such as proliferation, differentiation, and morphogenesis, in specific stages of development is crucial for proper self-organization of the embryo. Many of these processes have been investigated using actual embryos as model systems. However, mammalian development is difficult to explore *in vivo* due to the intra-uterine development; therefore, *ex vivo* culture methods have been developed. Even though these culture methods allow the investigation of embryonic development, culturing mammalian embryos *ex vivo* poses various limitations: First, mammals produce low numbers of embryos, impeding high-throughput screens. Second, generating genetically modified mammals is time-consuming and has low throughput. Third, culturing whole mammalian embryos *ex vivo* is not possible for all stages comparably (Nowotschin et al., 2019a; Aguilera-Castrejon et al., 2021). Furthermore, investigation of human development beyond 14 days is currently restricted due to ethical reasons (Rivron et al., 2018a; Hyun et al., 2021). Therefore, *in vitro* stem-cell-based models of development can help to overcome these limitations.

### Embryonic Development

Once oocyte and sperm fuse, the pre-implantation phase begins. Cells of the embryo divide until the 16-cell stage via cell cleavages [mouse embryonic day (E)3.0, human E4.0]. The first lineage segregations will result in the formation of a spherical structure with a central lumen, called the blastocyst (mouse E3.5, human E5.0). The outer layer of blastocysts consists of trophoblast cells, which will form extraembryonic tissue. The inner cell mass (ICM) of blastocysts will differentiate into epiblast (EPI) and primitive endoderm (PE) (mouse E4.5 and human E6.0), giving rise to the embryo and extraembryonic parietal and visceral endoderm, respectively. The latter will eventually form the yolk sac and embryonic endoderm. These mark some of the first critical differentiation processes occurring in embryogenesis (Rossant and Tam, 2009).

After the blastocyst has implanted into the uterus (mouse E5.0, human E9.0), epiblast cells give rise to the three germ layers: endoderm, mesoderm, and ectoderm, as well as primordial germ cells (PGCs). This process is known as gastrulation (mouse E6.5, human E17.0), referring to the observed *invagination* (Lim and Thiery, 2012). Gastrulation is followed by organogenesis (mouse E8.0, human E20.0), in which each germ layer will develop further into multiple tissue types. For instance, neural plate and neural tube form from the ectoderm (Nikolopoulou et al., 2017), while part of the mesoderm segments into blocks, known as somites, which will give rise to, for instance, muscle, skeleton, and dermis (Hubaud and Pourquie, 2014).

### Pluripotent Stem Cell Models

**FIGURE 1:**
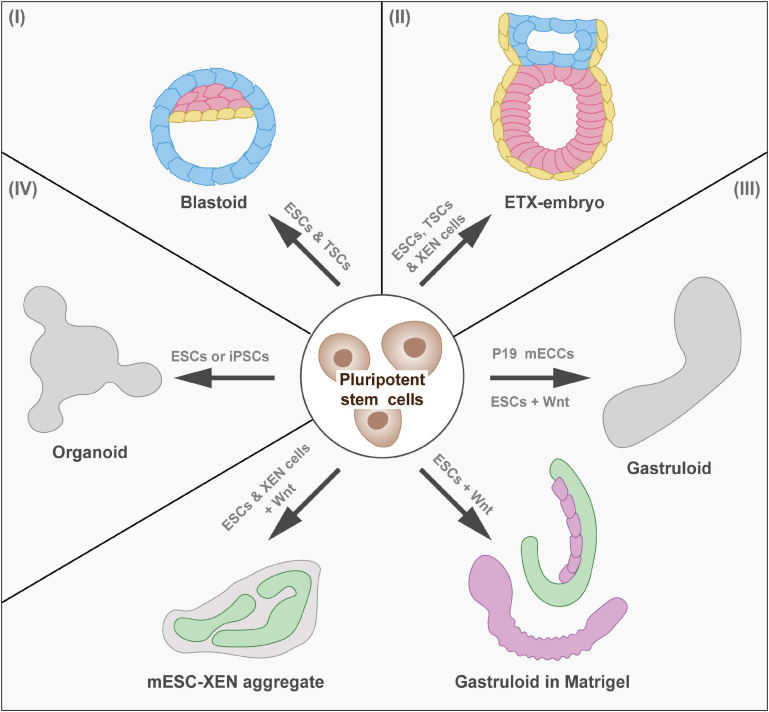
*In Vitro* models of mammalian development. Overview of model systems of pre-implantation and post-implantation development (I) Pre-implantation development: Blastoids model the blastocyst stage (mouse E3.5, human E5.0). (II) Peri-implantation and post-implantation development: ETX embryos model implantation and early stages of gastrulation (mouse E6.5, human E17.0). (III) Post-implantation development: Gastruloids show axial elongation and form cell types of the three germ layers (mouse E8, human E20.0). When embedded in Matrigel, gastruloids form segments and tubular structures resembling the posterior end of the mouse embryo. Adding extraembryonic endoderm (XEN) cells to ESCs during the gastruloid protocol results in the formation of neural tube-like structures, which recapitulate neural induction during development. (IV) Organogenesis (mouse E8.0, human E20.0): Developmental trajectories can be followed *in vitro* to generate organoid models of for instance the intestine. ESCs, embryonic stem cells; TSCs, trophoblast stem cells; EPS, extended pluripotent stem cells; ETX, embryonic stem cells, trophoblast stem cells, and extraembryonic endoderm cells; cAMP, cyclic adenosine monophosphate; ECCs, embryo carcinoma cells; iPSCs, induced pluripotent stem cells.

In light of recent advances in the study of molecular mechanisms during embryonic development using *in vitro* model systems (Figure 1), we outline and discuss such cultures and their use in developmental biology research. We particularly focus on models using pluripotent stem cells that recapitulate pre-implantation, peri-implantation, and post-implantation development of the whole embryo, as well as specific developmental trajectories toward parts of the embryo. Pluripotent stem cells can either be derived from the epiblast as embryonic stem cells (ESCs) (Evans and Kaufman, 1981) or they can be obtained as induced pluripotent stem cells (iPSCs) by reprogramming somatic cells (Takahashi and Yamanaka, 2006). Such *in vitro* model systems hold the potential to open new research avenues for the study of embryonic development and allow the study of human development.

## *In Vitro* Models of Mammalian Embryonic Development

### Pre-Implantation Model Systems

To model early embryonic development, including the first lineage segregations *in vitro*, blastoids have been established (Rivron et al., 2018b; Figure 1). Mouse ESCs were aggregated and overlaid with trophoblast stem cells (TSCs) in blastoid media, containing cyclic adenosine monophosphate (cAMP) and Wnt. These aggregates formed structures with blastocyst-like morphology that contained early blastocyst lineages and recapitulated aspects of implantation and decidua formation when implanted *in utero* (Rivron et al., 2018b). Variations to this protocol are the use of primed ESCs or extended pluripotent stem (EPS) cells for blastoid formation (Kime et al., 2019; Li et al., 2019; Sozen et al., 2019). Whereas primed ESCs recapitulate an epiblast-like cell state (Kime et al., 2016), EPS cells have been shown to contribute to both embryonic and extraembryonic lineages (Yang et al., 2017). While blastoids recapitulated early steps of implantation, *in utero* culture resulted in malformed structures and/or resorption (Rivron et al., 2018b; Kime et al., 2019; Li et al., 2019; Sozen et al., 2019). This could be due to slight variations in gene expression patterns of the involved cells (Posfai et al., 2021). Attempts to improve blastoid formation and post-implantation development are the optimization of TSCs prior to blastoid formation (Frias-Aldeguer et al., 2019) and the targeted modulation of signaling pathways during blastoid formation (Vrij et al., 2019). In future, protocols should be refined further to reflect morphology, cell differentiation, and epigenetic background of the blastocyst even more closely. Recently, protocols for the generation of human blastoids have been published (Liu et al., 2021; Yu et al., 2021). Even though efficiency is still low, this constitutes the first step toward studying human pre-implantation development.

Due to the high number with which blastoids can be generated, and the possibility for genetic manipulation, blastoids can serve as model systems to study lineage segregation, cellular mechanics, the process of implantation, and the effect of epigenetic abnormalities on early embryonic development. For instance, comparing mouse blastoids to troposphere, 3D cultures of TSCs, revealed pathways that signal between the epiblast and the trophoblast. Specifically, embryonic signals were identified that regulate proliferation, self-renewal, and epithelial morphogenesis of the trophectoderm enabling implantation *in utero* (Rivron et al., 2018b).

### Post-Implantation Model Systems

A 3D embryonic model mimicking development and morphology of the mouse embryo after implantation is the *ETS (ESC and TSC) embryos* (Harrison et al., 2017). Aggregates of ESCs and TSCs within an extracellular matrix form egg-shaped structures, which undertake symmetry breaking and form both nascent mesodermal cells and PGCs. To model development of later stages, including the morphogenetic changes of gastrulation, an improved 3D embryonic system has been developed. Since derivatives of PE cells were missing in ETS embryos, ESCs and TSCs were co-cultured with extraembryonic endoderm (XEN) cells, which resulted in the formation of *ETX (ESC, TSC and XEN cells) embryos* (Sozen et al., 2018). This model underwent gastrulation, even in the absence of extracellular matrix and recapitulated development until mouse E7.0, but not beyond this stage. Therefore, ETS and ETX embryos are ideally suited to study peri-implantation and early post-implantation development (Figure 1).

To recapitulate later stages of post-implantation development *in vitro*, researchers have focused on modeling parts of the embryo, which were often based on cultures of ESCs. Aggregated ESCs in differentiation-competent medium form *embryoid bodies*, which contain cells of all three germ layers. This model has, for instance, been used by ten Berge et al. (2008) to study the effect of Wnt signaling on self-organization and axis formation. Another structure to investigate Wnt signaling and axis elongation has been introduced by Marikawa et al. (2009). They showed that aggregates of mouse P19 embryo carcinoma cells (ECCs) exhibited mesoderm formation and axial elongation, which allowed the efficient study of molecular events controlling cell fate decisions (Figure 1). Based on such findings, *gastruloids* have been developed by van den Brink et al. (2014). They found that Wnt activation at a specific time after aggregation of ESCs induced symmetry breaking and axial extension, resulting in the formation of elongating structures containing cell types of all germ layers (Figure 1). While neuromesodermal progenitors (NMPs) formed at one pole of gastruloids (resembling the posterior side), PGCs, cardiac, endothelial, and head mesenchymal cells were located more anteriorly (Beccari et al., 2018). Furthermore, gastruloids have been shown to recapitulate the periodic segmentation of mammalian embryos, a process called somitogenesis, including the signaling dynamics of the segmentation clock (van den Brink et al., 2020).

Despite the presence of various cell types in gastruloids, such as neural tube, gastrointestinal tract, or pre-somatic cells (van den Brink et al., 2020), many processes of organogenesis do not occur spontaneously. Embedding gastruloids in a low percentage of extracellular matrix was found to result in a more defined morphology (van den Brink et al., 2020). Under these culture conditions, gastruloids elongated and formed physical segments resembling somites and occasionally tubular structures (Figure 1). Optimized culture conditions resulted in the development of a central neural tube with bilateral formation of somite-like assemblies and gut-like structures (Veenvliet et al., 2020). These gastruloids, remarkably similar in shape and organization to the posterior end of embryos, are referred to as *trunk-like structures* (Figure 1). To compensate for the absence of extraembryonic tissue in gastruloids, Bérenger-Currias et al. (2020) modified the gastruloid protocol by aggregating both mESCs and XEN cells. These aggregates induced neural tube-like structures that have a neural progenitor-like transcriptome and were shown to recapitulate neural tube development (Figure 1).

Moreover, models of human gastrulation have recently been developed. Simunovic et al. (2019) used human ESCs to model epiblast structures that break symmetry. Zheng et al. (2019) cultured human ESC and iPSC on extracellular material to model epiblast and amniotic ectoderm. These structures contained PGCs and primitive streak cells. Additionally, a protocol for the generation of human gastruloids has been published (Moris et al., 2020). Human gastruloids are elongating structures resembling aspects of Carnegie stage nine human embryos. Even though human gastruloids recapitulate later stages of post-implantation development, using this model does not raise ethical concerns of human embryo models, since anterior neural lineages were not present in these structures.

Post-implantation model systems have been used in several studies to dissect the mechanism of embryonic development. They are powerful models not only due to their close resemblance to the gastrulating embryo, but also because they can be used for high-throughput screens (van den Brink et al., 2020). Researchers have already utilized them to investigate signaling, cell fate decisions, self-organization, and stem cell niches in the gastrulating embryo (Turner et al., 2017; Martyn et al., 2019; Sagy et al., 2019).

## Recapitulating Selected Developmental Trajectories *In Vitro*

Over the last decades, researchers have made use of emerging knowledge on developmental trajectories from pluripotent stem cells toward certain cell lineages to guide *in vitro* differentiation along these trajectories. Cell differentiation can be induced in adherent 2D cultures to generate uniform cell populations. Akin to 3D structures derived from adult stem cells, known as *organoids*, pluripotent cells can be transferred to a 3D matrix during the differentiation process to allow self-organization of more complex structures (Eiraku et al., 2011). Recently, several protocols have been established applying this strategy to models developing organs *in vitro* (Figure 1). The established *in vitro* models of organ development will allow high-throughput screens and straightforward external perturbation for functional analysis. Here, we focus on *in vitro* model systems of the developing heart, gastrointestinal tract, and neural tube as examples of mesodermal, endodermal, and ectodermal derivatives.

### *In Vitro* Models of Heart Development

One of the first organs to form during embryogenesis is the heart, which originates from the lateral plate mesoderm. First, endocardial tubes merge to form the primitive heart tube. This then folds into shape and undergoes partitioning into four chambers (Haack and Abdelilah-Seyfried, 2016). To study cardiac biology and regeneration, cardiac organoid models have been established (Voges et al., 2017). A protocol has been developed in which the resulting organoids resemble mouse fetal hearts (Lee et al., 2020). In this protocol, mouse ESCs were aggregated in the presence of laminin and fibroblast growth factor (FGF) 4, which preceded incubation with a Wnt activator, bone morphogenetic factor (BMP) 4, and leukemia inhibitory factor. After 10 days, beating heart organoids containing cardiac chambers were observed. Another protocol for the generation of human fetal heart models consists of the aggregation of ESCs or iPSCs, which are exposed to two consecutive pulses of Wnt activation (Israeli et al., 2020). The resulting structures obtained fetal heart fate, mimicked heart development, and displayed chamber formation and vascularization. Although the presence of essential cell types was confirmed, the functionality of the vascularization still has to be analyzed in future studies.

Even though cardiac cells have been detected in various ESC-derived embryo-like models, such as embryoid bodies and gastruloids, cardiac morphology has been unreproducible in these model systems. Recently, gastruloids have been induced to form heart-like structures. Rossi et al. (2021) have modified the gastruloid protocol by adding cardiogenic factors, like basic FGF (bFGF), ascorbic acid, and vascular endothelial growth factor, to the culture medium after 4 days of culture. The resulting cardiac-like structures showed rhythmic calcium spiking, embryo-like beating rates, gene expression as seen in cardiac development, vascular-like compartment, and first and second heart field development. How multi-axial patterning and tissue communication, as seen in the embryo, impact morphogenetic processes of heart development has to be investigated in the future.

### *In Vitro* Models of Gastrointestinal Development

The gastrointestinal tract is derived from the endoderm, which first differentiates into the primitive gut. The primitive gut patterns into the foregut, midgut, and hindgut. These will then give rise to multiple organs of the gastrointestinal tract. The stomach, liver, and pancreas are formed from the foregut, whereas the appendix, colon, and rectum develop from the midgut and hindgut (Nowotschin et al., 2019b). *In vitro*, iPSCs can be guided along this differentiation trajectory to induce the generation of intestinal tissue, which form organoids when placed into extracellular matrix (Spence et al., 2011). Recently, a model has been introduced in which hepato-biliary-pancreatic (HBP) organ domains formed by co-culturing anterior and posterior gut spheroids (Koike et al., 2019). While FGF4 and Wnt promoted posterior gut fate in human pluripotent stem cells (hPSCs), BMP inhibition induced anterior gut fate. The two aggregates were then fused and embedded in Matrigel. Immunofluorescence staining and gene expression analysis confirmed multi-endoderm domains and HBP progenitor fate in this model system. Later stage processes, such as liver budding, has not been observed but might be induced by adding stromal cell components in the future. This is an example of how development can be guided *in vitro* along the *in vivo* developmental trajectories to form specific parts of the gastrointestinal tract (Wells and Spence, 2014).

### *In Vitro* Models of Neural Tube Development

**FIGURE 2:**
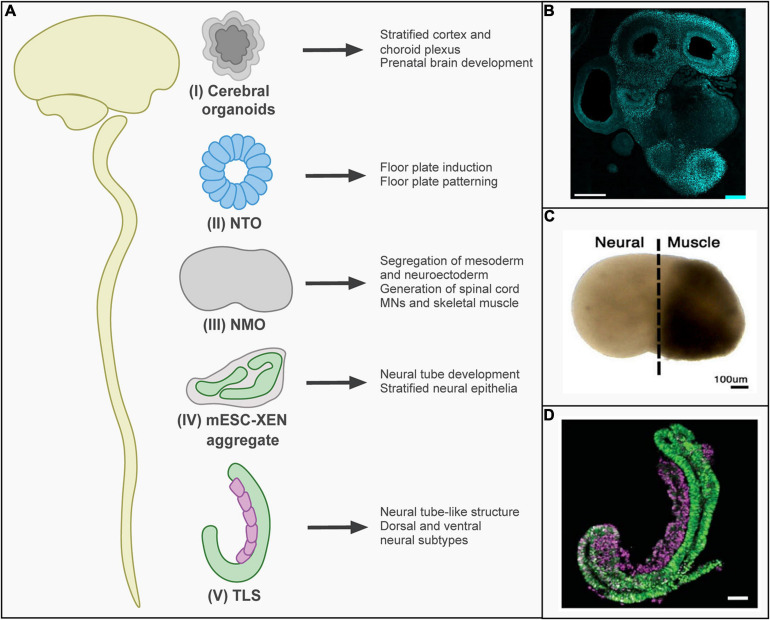
*In vitro* models of neural tube development. **(A)** *In vitro* stem-cell-based models reflecting different stages of neural tube development have been established: (I) Cerebral organoids recapitulate the cortex and choroid plexus of the brain. (II) Neural tube organoids (NTOs) have been used to examine the role of mechanistic forces on floor plate induction and patterning. (III) Neuromuscular organoids (NMOs) recapitulate thoracic/lumbar neural tube development. In the first 5 days, a segregation between neuroectoderm and mesoderm region occurs. At day 20, motor neurons (MNs) and myoblasts were present. (IV) mESC-XEN aggregates consist of stratified neural epithelia and were shown to follow *in vivo* mouse development. (V) Trunk-like structures (TLS) recapitulate neural tube development and form neural tube-like structures that consist of dorsal and ventral neural subtypes. **(B)** Representative image of cerebral organoids showing immunostaining for FOXG1 to visualize differentiation toward forebrain identity (scale bar 500 μm) [reprinted from Renner et al. (2017), with permission from John Wiley and Sons]. **(C)** Representative image of neuromuscular organoids [reprinted from Faustino Martins et al. (2020), with permission from Elsevier]. **(D)** Representative image of trunk-like structures expressing markers for mesodermal (purple) and neural (green) tissue (scale bar 100 μm) [reprinted from Veenvliet et al. (2020), with permission from AAAS.).

The neural plate develops from the ectoderm and folds into the neural tube, which gives rise to the nervous system (Nikolopoulou et al., 2017). The posterior neural tube elongates further by the differentiation of NMPs (Tzouanacou et al., 2009). Several models of neural tube formation and extension have been developed, of which some recapitulate anterior neurogenesis and others posterior neurogenesis (Figure 2). When aggregates of hPSCs are induced to differentiate to neural tissue and then embedded in a 3D matrix, cerebral organoids form over the course of weeks (Eiraku et al., 2008). These recapitulate cerebral cortex development and have for instance been used to study the pathophysiology of microcephaly (Lancaster et al., 2013; Figure 2B). Furthermore, transient formation of the neural tube during development has been modeled by generating neural tube organoids (NTOs). When mESC were embedded in an extracellular matrix in a differentiation-permissive medium, they formed neural cysts expressing anterior neural tube markers (Meinhardt et al., 2014; Ranga et al., 2016). Anteroposterior and dorsoventral identity could be modulated by the activation of retinoic acid or sonic hedgehog signaling (Ranga et al., 2016). In addition, morphogenetic changes inducing neural tube closure were mimicked *in vitro*, which improved neural differentiation and patterning of NTOs derived from hPSCs (Fattah et al., 2020; Figure 2).

Transient activation of Wnt signaling during neural differentiation can induce the formation of posterior neural tube structures (Metzis et al., 2018). A model recapitulating spinal cord development with connected muscle tissue has been introduced, termed neuromuscular organoids (NMOs) (Faustino Martins et al., 2020; Figure 2C). For this model, hiPSCs were differentiated into NMPs. These were then aggregated in media containing bFGF, hepatocyte growth factor, and insulin-like growth factor. Transcriptomics revealed that day 5 NMOs resembled NMPs in developing embryos. By day 50, NMOs had matured into structures containing both spinal cord neurons and skeletal muscle, resembling functional neuromuscular junctions. While NMO formation encompassed the aggregation of NMPs, the growing NMOs did not resemble the morphology of the developing neural tube. Gastruloids and similar structures (see above) are therefore important models of posterior neural tube development (Figure 2D). As discussed above, gastruloids form neural tube-like structures when embedded in Matrigel or surrounded by a layer of extraembryonic cells (Bérenger-Currias et al., 2020; van den Brink et al., 2020; Veenvliet et al., 2020).

## Recapitulating Selected Developmental Trajectories *In Vitro*

Thus, protocols have been developed to recapitulate cardiogenesis, gastrointestinal tract formation, and neural tube development. Even though these models are successful in mimicking parts of the development of specific organs, they do not resemble the entire organ or embryo consisting of multiple organs. More recent attempts to co-culture different organoids aim at addressing this by bringing multiple “organs” together (Koike et al., 2019). In addition, embryo-like model systems resembling gastrulating embryos can be directed toward specific trajectories and allow organogenesis within a multi-tissue and multi-organ context (Bérenger-Currias et al., 2020; Rossi et al., 2021). While such models have to be optimized and developed further, they will be of great benefit for the investigation of organogenesis during embryonic development.

## Discussion

Over the last decade, embryo-like model systems have been established recapitulating various steps of embryonic development. Blastoids enable the investigation of the first steps of compartmentalization, lineage segregation, and implantation. To study the latter, we need to develop and optimize *in vitro* implantation systems for both embryos and embryo-like structures further. ETX embryos, gastruloids, and other post-implantation models allow the study of gastrulation mechanisms, cellular dynamics, and signaling pathways coordinating the process of self-organization. *In vitro* models of organogenesis, such as cardiac organoids, hepato-biliary-pancreatic organoids, and neural tube organoids mimic aspects of the *in vivo* counterpart. *In vitro* models enable not only the study of embryonic development but also prenatal defects and the intrinsic regeneration potential of tissues. Once *in vitro* model systems are generated reproducibly in high numbers, they allow for high-throughput screens to develop stem cell or drug therapies. In addition, the investigation of human embryonic development in mechanistic detail will be possible. Despite these exciting advances and possibilities, it has to be stressed that the *in vivo* embryo will ultimately be the reference for these studies.

## Author Contributions

Both authors conceptualized and wrote the manuscript.

## Conflict of Interest

The authors declare that the research was conducted in the absence of any commercial or financial relationships that could be construed as a potential conflict of interest.
